# Di-μ-bromido-bis­({bis­[2-(2-pyrid­yl)eth­yl]amine}copper(II)) bis­(perchlorate)

**DOI:** 10.1107/S1600536807068663

**Published:** 2008-01-09

**Authors:** Ray J. Butcher, Yilma Gultneh, Teshome B. Yisgedu, Yohannes T. Tesema

**Affiliations:** a Department of Chemistry, Howard University, 525 College Street NW, Washington, DC 20059, USA

## Abstract

Each Cu atom in the dinuclear centrosymmetric title complex, [Cu_2_Br_2_(C_14_H_17_N_3_)_2_](ClO_4_)_2_, is ligated in a distorted square-pyramidal geometry (τ = 0.31) by a tridentate bis­[2-(2-pyrid­yl)eth­yl]amine ligand, and by two bridging Br atoms. In addition, the dinuclear species is stabilized by two hydrogen-bonded perchlorate anions.

## Related literature

For related literature, see: Chakrabarty *et al.* (2004 ▶); Helis *et al.* (1977 ▶); Marsh *et al.* (1983 ▶); Udugala-Ganehenege, *et al.* (2001 ▶); Xu *et al.* (2000 ▶). For the calculation of the coordination geometry, see: Addison *et al.* (1984 ▶).
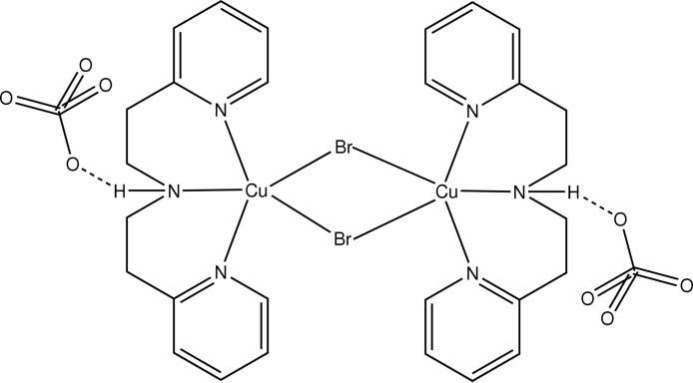


## Experimental

### 

#### Crystal data

[Cu_2_Br_2_(C_14_H_17_N_3_)_2_](ClO_4_)_2_
*M* *_r_* = 940.41
Triclinic, 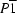

*a* = 6.8002 (13) Å
*b* = 11.413 (2) Å
*c* = 12.668 (2) Å
α = 67.212 (8)°
β = 77.019 (13)°
γ = 87.033 (15)°
*V* = 882.6 (3) Å^3^
*Z* = 1
Mo *K*α radiation
μ = 3.67 mm^−1^
*T* = 293 (2) K
0.42 × 0.21 × 0.18 mm

#### Data collection

Bruker P4 diffractometer
Absorption correction: ψ scan (North *et al.*, 1968 ▶) *T*_min_ = 0.569, *T*_max_ = 0.948 (expected range = 0.310–0.516)
3951 measured reflections
3936 independent reflections
2960 reflections with *I* > 2σ˘*I*)
*R*_int_ = 0.018
3 standard reflections every 97 reflections intensity decay: <2%

#### Refinement

*R*[*F* ^2^ > 2σ(*F* ^2^)] = 0.042
*wR*(*F* ^2^) = 0.095
*S* = 1.04
3936 reflections
255 parameters
50 restraints
H-atom parameters constrained
Δρ_max_ = 0.50 e Å^−3^
Δρ_min_ = −0.36 e Å^−3^

### 

Data collection: *XSCANS* (Bruker, 1997 ▶); cell refinement: *XSCANS*; data reduction: *XSCANS*; program(s) used to solve structure: *SHELXS97* (Sheldrick, 2008 ▶); program(s) used to refine structure: *SHELXL97* (Sheldrick, 2008 ▶); molecular graphics: *SHELXTL* (Bruker, 2000 ▶); software used to prepare material for publication: *SHELXTL*.

## Supplementary Material

Crystal structure: contains datablocks I, global. DOI: 10.1107/S1600536807068663/tk2238sup1.cif

Structure factors: contains datablocks I. DOI: 10.1107/S1600536807068663/tk2238Isup2.hkl

Additional supplementary materials:  crystallographic information; 3D view; checkCIF report

## Figures and Tables

**Table 1:** Hydrogen-bond geometry (Å, °)

*D*—H⋯*A*	*D*—H	H⋯*A*	*D*⋯*A*	*D*—H⋯*A*
N—H0*A*⋯O14*A*	0.91	2.34	3.205 (12)	159
N—H0*A*⋯O13*B*	0.91	2.44	3.089 (18)	129
C6*A*—H6*AB*⋯Br^i^	0.97	2.70	3.588 (4)	153
C6*B*—H6*BC*⋯Br^ii^	0.97	2.89	3.706 (4)	142
C6*B*—H6*BB*⋯O13*B*^iii^	0.97	2.57	3.487 (18)	158
C2*A*—H2*AA*⋯O11*A*^iv^	0.93	2.52	3.162 (14)	126
C7*A*—H7*AA*⋯O12*A*^v^	0.97	2.51	3.322 (16)	141
C7*A*—H7*AA*⋯O13*B*	0.97	2.49	3.179 (15)	128
C3*A*—H3*AA*⋯O13*A*^vi^	0.93	2.54	3.142 (12)	122

Symmetry codes: (i) 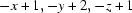; (ii) 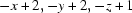; (iii) 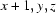; (iv) 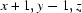; (v) 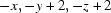; (vi) 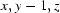.

**Fig. 1.**
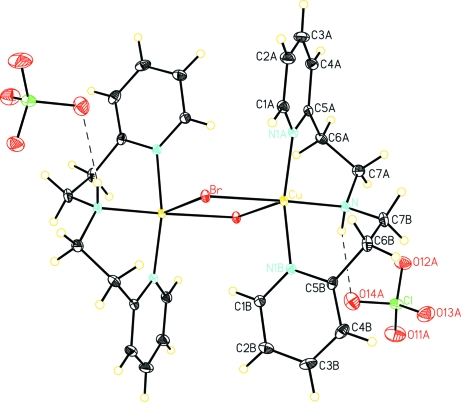
Complex (I) showing numbering scheme and displacement ellipsoids at the 20% probabilty level.

**Fig. 2.**
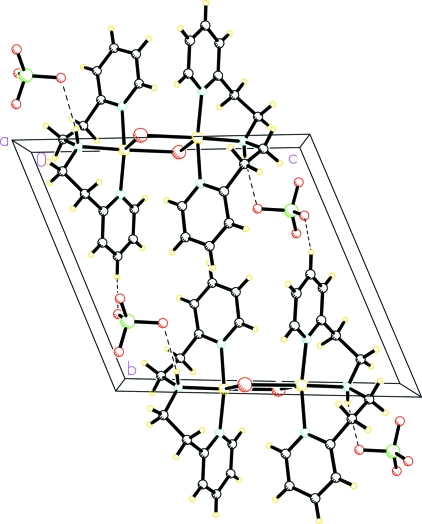
The packing arrangement viewed down the a axis showing the intramolecular N—H···O and intermolecular C—H···O interactions (dashed bonds).

## supplementary crystallographic information

### Comment

Complex (I), Fig. 1, contains two Cu(II) atoms, each within a distorted square-pyramidal geometry (τ = 0.31, Addison *et al.*, 1984) where one amine-N atom, two pyridine-N atoms and one Br atom constitute the basal plane with Cu—N_pyridine_ = 2.012 (3) and 2.000 (3) Å, Cu—N_amine_ = 2.044 (3) Å and Cu—Br = 2.4542 (7) Å. The axial position is occupied by the second Br atom with Cu—Br = 2.8908 (8) Å, the longer distance being consistent with a Jahn-Teller elongation. Pairs of these square-pyramidal Cu complexes form dimers about a center of inversion, being mutually bridged by the Br atoms. In addition, the dinuclear complex is stabilized by two N—H···O hydrogen bonded ClO_4_^-^ anions (Table 1) and the crystal packing is consolidated by a variety of hydrogen bonding interactions (Fig. 2 and Table 1).

### Experimental

The title complex was synthesized by reacting Cu(ClO_4_)_2_.6H_2_O (0.37 g, 1 mmol), bis[2-(2-pyridyl)ethyl]amine (0.227 g, 1 mmol) and potassium bromide (0.0297 g, 0.25 mmol) in acetonitrile (15 ml) for 4 h at room temperature. X-ray quality crystals were grown by slow diffusion of diethyl ether into an acetonitrile solution of the complex.

### Refinement

The perchlorate anion is disordered over two conformations with occupancy factors, from refinement, of 0.543 (17) and 0.457 (17). It was constrained to adopt a tetrahedral geometry. The H atoms were idealized with N—H = 0.91 Å and C—H = 0.93 (aromatic C—H), 0.96 (CH_3_), and 0.97 (CH_2_) Å, and with *U*_iso_(H) = 1.2*U*_eq_(C) (1.5*U*_eq_(C) for the CH_3_).

### Crystal data

**Table:**

[Cu_2_Br_2_(C_14_H_17_N_3_)_2_](ClO_4_)_2_	*Z* = 1
*M**_r_* = 940.41	*F*_000_ = 470
Triclinic, *P*1	*D*_x_ = 1.769 Mg m^−^^3^
Hall symbol: -P 1	Mo *K*α radiation λ = 0.71073 Å
*a* = 6.8002 (13) Å	Cell parameters from 49 reflections
*b* = 11.413 (2) Å	θ = 2.1–12.5º
*c* = 12.668 (2) Å	µ = 3.68 mm^−^^1^
α = 67.212 (8)º	*T* = 293 (2) K
β = 77.019 (13)º	Thick needle, blue
γ = 87.033 (15)º	0.42 × 0.21 × 0.18 mm
*V* = 882.6 (3) Å^3^	

### Data collection

**Table:**

Bruker P4 diffractometer	*R*_int_ = 0.018
Radiation source: fine-focus sealed tube	θ_max_ = 27.5º
Monochromator: graphite	θ_min_ = 2.1º
*T* = 293(2) K	*h* = −8→0
ω scans	*k* = −13→13
Absorption correction: ψ scan(North *et al.*, 1968)	*l* = −16→16
*T*_min_ = 0.569, *T*_max_ = 0.948	3 standard reflections
3951 measured reflections	every 97 reflections
3936 independent reflections	intensity decay: <2%
2960 reflections with *I* > 2σ˘*I*)	

### Refinement

**Table:**

Refinement on *F*^2^	Secondary atom site location: difference Fourier map
Least-squares matrix: full	Hydrogen site location: inferred from neighbouring sites
*R*[*F*^2^ > 2σ(*F*^2^)] = 0.042	H-atom parameters constrained
*wR*(*F*^2^) = 0.095	*w* = 1/[σ^2^(*F*_o_^2^) + (0.0336*P*)^2^ + 0.6582*P*] where *P* = (*F*_o_^2^ + 2*F*_c_^2^)/3
*S* = 1.04	(Δ/σ)_max_ < 0.001
3936 reflections	Δρ_max_ = 0.50 e Å^−^^3^
255 parameters	Δρ_min_ = −0.36 e Å^−^^3^
50 restraints	Extinction correction: none
Primary atom site location: structure-invariant direct methods	

### Special details

**Table:**

Geometry. All e.s.d.'s (except the e.s.d. in the dihedral angle between two l.s. planes) are estimated using the full covariance matrix. The cell e.s.d.'s are taken into account individually in the estimation of e.s.d.'s in distances, angles and torsion angles; correlations between e.s.d.'s in cell parameters are only used when they are defined by crystal symmetry. An approximate (isotropic) treatment of cell e.s.d.'s is used for estimating e.s.d.'s involving l.s. planes.
Refinement. Refinement of *F*^2^ against ALL reflections. The weighted *R*-factor *wR* and goodness of fit *S* are based on *F*^2^, conventional *R*-factors *R* are based on *F*, with *F* set to zero for negative *F*^2^. The threshold expression of *F*^2^ > σ(*F*^2^) is used only for calculating *R*-factors(gt) *etc*. and is not relevant to the choice of reflections for refinement. *R*-factors based on *F*^2^ are statistically about twice as large as those based on *F*, and *R*- factors based on ALL data will be even larger.

### Fractional atomic coordinates and isotropic or equivalent isotropic displacement parameters (Å^2^)

**Table:**

	*x*	*y*	*z*	*U*_iso_*/*U*_eq_	Occ. (<1)
Cu	0.60810 (7)	0.98360 (4)	0.63720 (4)	0.03731 (14)	
Br	0.76316 (6)	0.97756 (4)	0.44483 (3)	0.03934 (12)	
Cl	0.19832 (18)	1.27274 (11)	0.86706 (12)	0.0588 (3)	
O11A	0.062 (3)	1.3663 (19)	0.856 (2)	0.149 (8)	0.543 (17)
O12A	0.117 (3)	1.1516 (12)	0.9388 (14)	0.094 (5)	0.543 (17)
O13A	0.372 (2)	1.2975 (11)	0.8979 (15)	0.110 (4)	0.543 (17)
O14A	0.2714 (18)	1.2648 (13)	0.7496 (8)	0.101 (4)	0.543 (17)
O11B	0.010 (2)	1.332 (2)	0.8582 (19)	0.100 (5)	0.457 (17)
O12B	0.346 (3)	1.3175 (18)	0.7719 (15)	0.155 (7)	0.457 (17)
O13B	0.155 (3)	1.1399 (11)	0.9066 (16)	0.072 (4)	0.457 (17)
O14B	0.256 (3)	1.2875 (13)	0.9664 (14)	0.110 (5)	0.457 (17)
N	0.4740 (5)	0.9948 (3)	0.7943 (3)	0.0424 (8)	
H0A	0.3920	1.0619	0.7755	0.051*	
N1A	0.6270 (5)	0.7958 (3)	0.7201 (3)	0.0391 (7)	
N1B	0.6733 (5)	1.1709 (3)	0.5811 (3)	0.0380 (7)	
C1A	0.8000 (7)	0.7390 (4)	0.6945 (4)	0.0495 (10)	
H1AA	0.9093	0.7891	0.6398	0.059*	
C2A	0.8210 (9)	0.6113 (5)	0.7456 (5)	0.0666 (14)	
H2AA	0.9433	0.5751	0.7279	0.080*	
C3A	0.6575 (9)	0.5370 (5)	0.8238 (5)	0.0730 (16)	
H3AA	0.6662	0.4492	0.8572	0.088*	
C4A	0.4811 (8)	0.5932 (4)	0.8524 (4)	0.0581 (12)	
H4AA	0.3702	0.5436	0.9058	0.070*	
C5A	0.4694 (6)	0.7240 (4)	0.8012 (3)	0.0412 (9)	
C6A	0.2871 (6)	0.7913 (4)	0.8353 (4)	0.0487 (10)	
H6AA	0.1859	0.7293	0.8942	0.058*	
H6AB	0.2304	0.8359	0.7672	0.058*	
C7A	0.3377 (8)	0.8864 (4)	0.8842 (4)	0.0609 (13)	
H7AA	0.2134	0.9198	0.9144	0.073*	
H7AB	0.4022	0.8424	0.9492	0.073*	
C1B	0.6256 (7)	1.2605 (4)	0.4855 (4)	0.0488 (10)	
H1BA	0.5619	1.2350	0.4397	0.059*	
C2B	0.6661 (8)	1.3874 (4)	0.4520 (5)	0.0609 (13)	
H2BA	0.6339	1.4470	0.3842	0.073*	
C3B	0.7563 (8)	1.4244 (5)	0.5220 (6)	0.0681 (15)	
H3BA	0.7819	1.5103	0.5031	0.082*	
C4B	0.8084 (7)	1.3342 (5)	0.6198 (5)	0.0623 (14)	
H4BA	0.8725	1.3584	0.6662	0.075*	
C5B	0.7646 (6)	1.2071 (4)	0.6483 (4)	0.0435 (10)	
C6B	0.8117 (7)	1.1013 (5)	0.7541 (4)	0.0544 (12)	
H6BB	0.8924	1.1359	0.7902	0.065*	
H6BC	0.8917	1.0401	0.7290	0.065*	
C7B	0.6244 (7)	1.0334 (5)	0.8450 (4)	0.0546 (12)	
H7BB	0.6642	0.9582	0.9043	0.065*	
H7BC	0.5613	1.0889	0.8835	0.065*	

### Atomic displacement parameters (Å^2^)

**Table:**

	*U*^11^	*U*^22^	*U*^33^	*U*^12^	*U*^13^	*U*^23^
Cu	0.0479 (3)	0.0350 (3)	0.0253 (2)	−0.0001 (2)	−0.0020 (2)	−0.01082 (19)
Br	0.0328 (2)	0.0541 (3)	0.0300 (2)	0.00276 (17)	−0.00239 (15)	−0.01773 (18)
Cl	0.0517 (7)	0.0459 (6)	0.0707 (8)	0.0054 (5)	−0.0143 (6)	−0.0138 (6)
O11A	0.161 (13)	0.099 (11)	0.191 (11)	0.081 (10)	−0.070 (10)	−0.051 (9)
O12A	0.097 (8)	0.071 (7)	0.085 (8)	−0.005 (6)	0.011 (6)	−0.013 (5)
O13A	0.106 (8)	0.093 (6)	0.145 (10)	−0.012 (6)	−0.050 (7)	−0.047 (7)
O14A	0.080 (7)	0.125 (9)	0.080 (6)	0.007 (6)	0.004 (5)	−0.035 (5)
O11B	0.098 (8)	0.102 (11)	0.128 (9)	0.067 (8)	−0.054 (7)	−0.065 (8)
O12B	0.120 (10)	0.146 (11)	0.119 (10)	−0.020 (8)	0.044 (9)	0.000 (9)
O13B	0.066 (7)	0.050 (5)	0.109 (11)	0.007 (5)	−0.025 (7)	−0.037 (6)
O14B	0.146 (13)	0.105 (8)	0.104 (10)	−0.010 (9)	−0.058 (9)	−0.048 (8)
N	0.049 (2)	0.0437 (19)	0.0311 (17)	0.0068 (16)	−0.0029 (15)	−0.0148 (15)
N1A	0.047 (2)	0.0372 (17)	0.0296 (16)	0.0006 (15)	−0.0069 (14)	−0.0102 (14)
N1B	0.0389 (18)	0.0410 (18)	0.0323 (17)	−0.0025 (14)	−0.0032 (14)	−0.0143 (14)
C1A	0.049 (3)	0.051 (3)	0.044 (2)	0.007 (2)	−0.008 (2)	−0.016 (2)
C2A	0.074 (4)	0.052 (3)	0.062 (3)	0.018 (3)	−0.007 (3)	−0.015 (3)
C3A	0.097 (4)	0.037 (3)	0.069 (3)	0.009 (3)	−0.009 (3)	−0.010 (2)
C4A	0.071 (3)	0.043 (3)	0.045 (3)	−0.006 (2)	0.001 (2)	−0.007 (2)
C5A	0.050 (2)	0.042 (2)	0.0287 (19)	−0.0002 (18)	−0.0094 (17)	−0.0101 (17)
C6A	0.044 (2)	0.046 (2)	0.041 (2)	−0.0035 (19)	−0.0038 (19)	−0.0031 (19)
C7A	0.074 (3)	0.052 (3)	0.042 (2)	0.002 (2)	0.012 (2)	−0.016 (2)
C1B	0.055 (3)	0.046 (2)	0.045 (2)	−0.002 (2)	−0.012 (2)	−0.016 (2)
C2B	0.059 (3)	0.044 (3)	0.063 (3)	−0.003 (2)	−0.004 (2)	−0.007 (2)
C3B	0.058 (3)	0.043 (3)	0.097 (4)	−0.011 (2)	0.006 (3)	−0.032 (3)
C4B	0.049 (3)	0.073 (3)	0.079 (4)	−0.015 (2)	−0.004 (3)	−0.047 (3)
C5B	0.037 (2)	0.057 (3)	0.042 (2)	−0.0043 (19)	−0.0035 (18)	−0.026 (2)
C6B	0.045 (2)	0.083 (3)	0.045 (3)	0.012 (2)	−0.015 (2)	−0.033 (2)
C7B	0.060 (3)	0.074 (3)	0.032 (2)	0.011 (2)	−0.014 (2)	−0.021 (2)

### Geometric parameters (Å, °)

**Table:**

Cu—N1A	2.000 (3)	C3A—C4A	1.373 (7)
Cu—N1B	2.012 (3)	C3A—H3AA	0.9300
Cu—N	2.044 (3)	C4A—C5A	1.385 (6)
Cu—Br	2.4542 (7)	C4A—H4AA	0.9300
Cu—Br^i^	2.8908 (8)	C5A—C6A	1.494 (6)
Br—Cu^i^	2.8908 (8)	C6A—C7A	1.528 (7)
Cl—O12B	1.326 (11)	C6A—H6AA	0.9700
Cl—O11A	1.361 (11)	C6A—H6AB	0.9700
Cl—O12A	1.388 (11)	C7A—H7AA	0.9700
Cl—O13A	1.397 (9)	C7A—H7AB	0.9700
Cl—O13B	1.424 (11)	C1B—C2B	1.366 (6)
Cl—O11B	1.424 (11)	C1B—H1BA	0.9300
Cl—O14B	1.469 (10)	C2B—C3B	1.379 (8)
Cl—O14A	1.495 (9)	C2B—H2BA	0.9300
N—C7B	1.488 (6)	C3B—C4B	1.375 (8)
N—C7A	1.496 (5)	C3B—H3BA	0.9300
N—H0A	0.9100	C4B—C5B	1.384 (6)
N1A—C5A	1.349 (5)	C4B—H4BA	0.9300
N1A—C1A	1.350 (5)	C5B—C6B	1.502 (6)
N1B—C1B	1.342 (5)	C6B—C7B	1.517 (6)
N1B—C5B	1.349 (5)	C6B—H6BB	0.9700
C1A—C2A	1.361 (6)	C6B—H6BC	0.9700
C1A—H1AA	0.9300	C7B—H7BB	0.9700
C2A—C3A	1.374 (7)	C7B—H7BC	0.9700
C2A—H2AA	0.9300		
			
N1A—Cu—N1B	159.15 (14)	C5A—C4A—H4AA	120.2
N1A—Cu—N	89.40 (13)	N1A—C5A—C4A	120.4 (4)
N1B—Cu—N	85.66 (13)	N1A—C5A—C6A	117.6 (4)
N1A—Cu—Br	92.56 (9)	C4A—C5A—C6A	122.0 (4)
N1B—Cu—Br	92.79 (9)	C5A—C6A—C7A	111.7 (4)
N—Cu—Br	177.86 (10)	C5A—C6A—H6AA	109.3
N1A—Cu—Br^i^	106.74 (10)	C7A—C6A—H6AA	109.3
N1B—Cu—Br^i^	93.79 (10)	C5A—C6A—H6AB	109.3
N—Cu—Br^i^	93.61 (10)	C7A—C6A—H6AB	109.3
Br—Cu—Br^i^	85.00 (2)	H6AA—C6A—H6AB	107.9
Cu—Br—Cu^i^	95.00 (2)	N—C7A—C6A	112.9 (3)
O11A—Cl—O13A	113.7 (9)	N—C7A—H7AA	109.0
O12A—Cl—O13A	111.2 (9)	C6A—C7A—H7AA	109.0
O12B—Cl—O11B	115.9 (11)	N—C7A—H7AB	109.0
O13B—Cl—O11B	105.7 (9)	C6A—C7A—H7AB	109.0
O12B—Cl—O14B	110.1 (9)	H7AA—C7A—H7AB	107.8
O13B—Cl—O14B	104.4 (8)	N1B—C1B—C2B	123.3 (4)
O11B—Cl—O14B	106.0 (9)	N1B—C1B—H1BA	118.4
O11A—Cl—O14A	108.1 (10)	C2B—C1B—H1BA	118.4
O12A—Cl—O14A	103.5 (7)	C1B—C2B—C3B	117.9 (5)
O13A—Cl—O14A	105.2 (6)	C1B—C2B—H2BA	121.1
C7B—N—C7A	112.0 (3)	C3B—C2B—H2BA	121.1
C7B—N—Cu	110.9 (3)	C4B—C3B—C2B	119.9 (5)
C7A—N—Cu	118.5 (3)	C4B—C3B—H3BA	120.0
C7B—N—H0A	104.7	C2B—C3B—H3BA	120.0
C7A—N—H0A	104.7	C3B—C4B—C5B	119.3 (5)
Cu—N—H0A	104.7	C3B—C4B—H4BA	120.4
C5A—N1A—C1A	119.0 (4)	C5B—C4B—H4BA	120.4
C5A—N1A—Cu	121.3 (3)	N1B—C5B—C4B	120.9 (4)
C1A—N1A—Cu	119.8 (3)	N1B—C5B—C6B	115.5 (4)
C1B—N1B—C5B	118.7 (4)	C4B—C5B—C6B	123.6 (4)
C1B—N1B—Cu	124.8 (3)	C5B—C6B—C7B	113.2 (4)
C5B—N1B—Cu	116.4 (3)	C5B—C6B—H6BB	108.9
N1A—C1A—C2A	122.7 (4)	C7B—C6B—H6BB	108.9
N1A—C1A—H1AA	118.7	C5B—C6B—H6BC	108.9
C2A—C1A—H1AA	118.7	C7B—C6B—H6BC	108.9
C1A—C2A—C3A	118.5 (5)	H6BB—C6B—H6BC	107.8
C1A—C2A—H2AA	120.7	N—C7B—C6B	113.2 (3)
C3A—C2A—H2AA	120.7	N—C7B—H7BB	108.9
C4A—C3A—C2A	119.7 (5)	C6B—C7B—H7BB	108.9
C4A—C3A—H3AA	120.1	N—C7B—H7BC	108.9
C2A—C3A—H3AA	120.1	C6B—C7B—H7BC	108.9
C3A—C4A—C5A	119.6 (4)	H7BB—C7B—H7BC	107.7
C3A—C4A—H4AA	120.2		
			
N1A—Cu—Br—Cu^i^	106.59 (10)	N1A—C1A—C2A—C3A	1.8 (8)
N1B—Cu—Br—Cu^i^	−93.56 (10)	C1A—C2A—C3A—C4A	−2.8 (9)
N—Cu—Br—Cu^i^	−50 (3)	C2A—C3A—C4A—C5A	0.6 (8)
Br^i^—Cu—Br—Cu^i^	0.0	C1A—N1A—C5A—C4A	−3.7 (6)
N1A—Cu—N—C7B	97.3 (3)	Cu—N1A—C5A—C4A	176.0 (3)
N1B—Cu—N—C7B	−62.4 (3)	C1A—N1A—C5A—C6A	174.5 (4)
Br—Cu—N—C7B	−106 (3)	Cu—N1A—C5A—C6A	−5.8 (5)
Br^i^—Cu—N—C7B	−155.9 (3)	C3A—C4A—C5A—N1A	2.7 (7)
N1A—Cu—N—C7A	−34.3 (3)	C3A—C4A—C5A—C6A	−175.4 (5)
N1B—Cu—N—C7A	166.0 (3)	N1A—C5A—C6A—C7A	−58.6 (5)
Br—Cu—N—C7A	122 (3)	C4A—C5A—C6A—C7A	119.6 (5)
Br^i^—Cu—N—C7A	72.5 (3)	C7B—N—C7A—C6A	−142.0 (4)
N1B—Cu—N1A—C5A	121.2 (4)	Cu—N—C7A—C6A	−10.9 (5)
N—Cu—N1A—C5A	45.1 (3)	C5A—C6A—C7A—N	65.9 (5)
Br—Cu—N1A—C5A	−134.1 (3)	C5B—N1B—C1B—C2B	0.4 (7)
Br^i^—Cu—N1A—C5A	−48.5 (3)	Cu—N1B—C1B—C2B	177.7 (4)
N1B—Cu—N1A—C1A	−59.1 (5)	N1B—C1B—C2B—C3B	−1.5 (7)
N—Cu—N1A—C1A	−135.2 (3)	C1B—C2B—C3B—C4B	2.1 (8)
Br—Cu—N1A—C1A	45.6 (3)	C2B—C3B—C4B—C5B	−1.7 (8)
Br^i^—Cu—N1A—C1A	131.2 (3)	C1B—N1B—C5B—C4B	0.1 (6)
N1A—Cu—N1B—C1B	157.2 (4)	Cu—N1B—C5B—C4B	−177.4 (3)
N—Cu—N1B—C1B	−126.0 (3)	C1B—N1B—C5B—C6B	179.6 (4)
Br—Cu—N1B—C1B	52.5 (3)	Cu—N1B—C5B—C6B	2.1 (4)
Br^i^—Cu—N1B—C1B	−32.6 (3)	C3B—C4B—C5B—N1B	0.6 (7)
N1A—Cu—N1B—C5B	−25.4 (5)	C3B—C4B—C5B—C6B	−178.9 (4)
N—Cu—N1B—C5B	51.3 (3)	N1B—C5B—C6B—C7B	−66.2 (5)
Br—Cu—N1B—C5B	−130.2 (3)	C4B—C5B—C6B—C7B	113.3 (5)
Br^i^—Cu—N1B—C5B	144.7 (3)	C7A—N—C7B—C6B	156.6 (4)
C5A—N1A—C1A—C2A	1.4 (7)	Cu—N—C7B—C6B	21.7 (5)
Cu—N1A—C1A—C2A	−178.3 (4)	C5B—C6B—C7B—N	49.4 (5)

Symmetry codes: (i) −*x*+1, −*y*+2, −*z*+1.

### Hydrogen-bond geometry (Å, °)

**Table:**

*D*—H···*A*	*D*—H	H···*A*	*D*···*A*	*D*—H···*A*
N—H0A···O14A	0.91	2.34	3.205 (12)	159
N—H0A···O13B	0.91	2.44	3.089 (18)	129
C6A—H6AB···Br^i^	0.97	2.70	3.588 (4)	153
C6B—H6BC···Br^ii^	0.97	2.89	3.706 (4)	142
C6B—H6BB···O13B^iii^	0.97	2.57	3.487 (18)	158
C2A—H2AA···O11A^iv^	0.93	2.52	3.162 (14)	126
C7A—H7AA···O12A^v^	0.97	2.51	3.322 (16)	141
C7A—H7AA···O13B	0.97	2.49	3.179 (15)	128
C3A—H3AA···O13A^vi^	0.93	2.54	3.142 (12)	122

Symmetry codes: (i) −*x*+1, −*y*+2, −*z*+1; (ii) −*x*+2, −*y*+2, −*z*+1; (iii) *x*+1, *y*, *z*; (iv) *x*+1, *y*−1, *z*; (v) −*x*, −*y*+2, −*z*+2; (vi) *x*, *y*−1, *z*.
